# Gemcitabine-docetaxel therapy in pediatric patients with relapsed or refractory sarcoma: a single-center experience

**DOI:** 10.3389/fped.2026.1762698

**Published:** 2026-01-26

**Authors:** Metin Çil, Begül Ganiye Yağcı

**Affiliations:** Division of Pediatric Hematology and Oncology, Department of Pediatrics, Adana City Training and Research Hospital, Ministry of Health, Adana, Türkiye

**Keywords:** DOCETAXEL, gemcitabine, GEMDOX, palliative chemotherapy, pediatric sarcoma, relapsed/refractory, soft tissue sarcoma

## Abstract

**Background:**

The prognosis for pediatric patients with relapsed or refractory sarcoma remains poor, and standard salvage therapies are lacking. This study evaluated the efficacy and toxicity of the gemcitabine and docetaxel (GEMDOX) combination in this patient population.

**Methods:**

We retrospectively analyzed 36 pediatric patients treated with GEMDOX at our institution between 2015 and 2025. Patients received gemcitabine (1,000 mg/m^2^ on days 1 and 8) and docetaxel (100 mg/m^2^ on day 8) in 21-day cycles.

**Results:**

The median age was 13.5 years, with osteosarcoma being the most common diagnosis (58.3%). GEMDOX was administered predominantly as a third-line regimen (58.3%). The objective response rate (ORR) at the final assessment was 5.8%, and the disease control rate (DCR) was 14.6%. The median progression-free survival (PFS) and overall survival (OS) were 5.72 months (95% CI, 3.95–7.48) and 12.33 months (95% CI, 8.97–15.66), respectively. The most common Grade 3–4 toxicities were neutropenia (22.2%) and febrile neutropenia (19.4%), both of which were manageable with G-CSF support. No treatment-related mortality occurred.

**Conclusions:**

Although the objective response rate was modest in this heavily pretreated cohort, GEMDOX demonstrated a manageable safety profile. It represents a viable palliative option with a manageable toxicity profile for pediatric patients with relapsed/refractory sarcoma when curative options are limited. However, given its intensive nature, optimal efficacy may be better achieved when utilized earlier in the relapse setting rather than as a late-line rescue therapy.

## Introduction

Sarcomas are malignant tumors of bone and soft tissue arising from mesenchymal cells. Although rare in childhood, they account for a significant portion of all pediatric malignancies. Indeed, according to the global analysis of the International Incidence of Childhood Cancer, Third Edition (IICC-3) covering data from 2001 to 2010, bone tumors (4.7%) and soft tissue sarcomas (4.9%) collectively account for approximately 9.6% of all cancers in children aged 0–14 year (1, 2). The most common sarcoma types are rhabdomyosarcoma, osteosarcoma, and Ewing sarcoma. Among these, rhabdomyosarcoma is the most common malignant soft tissue tumor of childhood, accounting for approximately 3% of all pediatric cancers. Osteosarcoma is the most frequent bone sarcoma, with an incidence of approximately 3.1 cases per million (2, 3). Ewing sarcoma is also common in the pediatric age group and typically arises in the pelvis, long bones, and chest wall. Other types, such as fibrosarcoma, synovial sarcoma, and malignant peripheral nerve sheath tumor, are rarer (3, 4).

The management of pediatric sarcomas is based on a multimodal approach that utilizes combinations of surgery, radiotherapy, and systemic chemotherapy. Standard treatment options include the methotrexate, doxorubicin, and cisplatin (MAP) regimen for osteosarcoma; regimens containing vincristine, doxorubicin, cyclophosphamide, ifosfamide, and etoposide (VDC-IE) for Ewing sarcoma; and the vincristine, actinomycin D, and cyclophosphamide (VAC) regimen for rhabdomyosarcoma (3).

Despite standard chemotherapy regimens, approximately 30% of patients will relapse, and in this setting, treatment options become even more limited. Even though there are different novel treatment options, the prognosis for patients with advanced-stage soft tissue and bone sarcoma remains poor, with overall survival rates ranging from 0% to 45% (4, 5). Adriamycin (doxorubicin)-based combinations are the most commonly used chemotherapy regimens, with response rates ranging from 25% to 40%. However, the median overall survival (OS) remains limited to only 8–12 months (6, 7). Agents such as high-dose ifosfamide, temozolomide, irinotecan, and topotecan are used in the salvage setting for relapsed and refractory sarcomas, but the progression-free survival (PFS) achieved with these regimens is generally short (3, 4). The poor outcomes in this challenging patient population necessitate the investigation of novel and effective chemotherapy combinations, such as gemcitabine and docetaxel (GEMDOX).

Gemcitabine is a nucleoside analog that inhibits DNA synthesis by affecting the cell in the S phase and is used in many solid tumors (5, 8) Docetaxel is a semi-synthetic taxane derivative that inhibits cell division in the G2/M phase by stabilizing microtubules (8, 9). Preclinical *in vitro* studies have demonstrated that the combination of gemcitabine and docetaxel exhibits synergistic and additive cytotoxic effects on numerous tumors, including the SAOS-2 (cultured osteosarcoma cell line) line (8). The GEMDOX combination was first evaluated in 34 adult patients with leiomyosarcoma who had received up to two prior chemotherapy regimens, showing significant antitumor activity with an objective response rate of 53% (10). In the pediatric patients, Navid et al. first studied this regimen in 2008 in 22 patients with refractory bone sarcoma, reporting an objective response rate of 29% (11). More recently, a larger multicenter phase II trial (SARC003) evaluated this combination in children and adults with recurrent bone sarcomas. In contrast to earlier reports, this study observed more modest activity, with objective response rates of less than 6% in patients with osteosarcoma and Ewing sarcoma, highlighting the variability in outcomes across different cohorts (12). The most common side effects of GEMDOX are hematologic toxicities, including neutropenia, anemia, and thrombocytopenia. In addition, nausea, extremity edema, skin rash, and hepatotoxicity have also been reported (5, 13) In conclusion, when choosing a salvage protocol for relapsed patients, the ideal therapy should be easy to administration and have manageable side effects. It is also crucial to use drugs with different mechanisms of action to overcome resistance from previous treatments. GEMDOX was chosen for this study because it fulfills these requirements as a non-cross-resistant alternative. However, more clinical studies are needed to determine which groups this combination is more effective and to investigate its long-term side effects (14). The primary objective of this study was to evaluate the efficacy, specifically the disease control rate, and the safety profile of the GEMDOX combination in our cohort, and to compare our findings with those reported in the literature.

### Patients and methods

We retrospectively reviewed the medical records of patients diagnosed with relapsed or refractory sarcoma over the last 10 years who received at least two cycles of gemcitabine and docetaxel at our institution (final data cutoff: March 31, 2025). The records were assessed for patient characteristics, disease diagnosis, prior therapies, concomitant therapies, treatment outcomes, and toxicity. The study was approved by our institution's Corporate Review Board with decision number 060325/423.

GEMDOX therapy was administered every 21 days. All patients received gemcitabine at a dose of 1,000 mg/m^2^ intravenously (IV) over 90 min on days 1 and 8, followed by docetaxel at a dose of 100 mg/m^2^ IV administered over 1 h on day 8. Patients were given ondansetron or granisetron as antiemetic prophylaxis and dexamethasone (or corticosteroid) premedication to prevent fluid retention and hypersensitivity reactions prior to chemotherapy on days 1 and 8. The G-CSF (Granulocyte-Colony Stimulating Factor) administration protocol was determined based on patient risk status. Patients considered high-risk (a history of sepsis, grade 4 hematologic toxicity during prior chemotherapy, or low bone marrow reserve) received primary prophylaxis. For all other patients, G-CSF was initiated as secondary prophylaxis if they experienced febrile neutropenia, Grade 3–4 neutropenia, or had their treatment initiation delayed for more than one week due to neutropenia in a prior cycle. When administered, G-CSF was initiated 24 h following day 8 of the chemotherapy cycle and continued until the absolute neutrophil count (ANC) increased above 1,000 /mm^3^. Chemotherapy was continued unless there was disease progression, intolerance to treatment, or the patient discontinued treatment. Before the administration of the next chemotherapy cycle, all toxicities from the last cycle were required to resolve to grade 1 or less. For patients who experienced a treatment delay of more than one week due to a grade 3 or 4 toxicity, a 20% dose reduction was applied to the subsequent cycle. Radiotherapy was administered sequentially and not concomitantly with gemcitabine to avoid the risk of severe toxicity due to the radiosensitizing effect of gemcitabine. Toxicity was graded according to the National Cancer Institute (NCI) Common Terminology Criteria for Adverse Events (CTCAE), version 5.0. An adverse event was recorded as a toxicity if the criteria were met at least once at any point during a treatment cycle.

The radiological evaluation of the tumor response to chemotherapy was assessed using Response Evaluation Criteria in Solid Tumor (RECIST) version 1.1. Disease response was classified as follows: 1) Complete response (CR) (disappearance of all lesions and no detection of new lesions), 2) Partial response (PR) (at least a 30% decrease in disease measurement and no detection of new lesions), 3) Progressive disease (PD) (at least a 20% increase in disease measurement or the appearance of a new lesion), 4) Stable disease (SD) (not classified in the CR, PR, or PD groups). These evaluation criteria were applied to lesions measurable by computed tomography (CT), defined as those having at least one dimension of ≥10 mm. For smaller lesions, a more subjective assessment was performed: lesions that disappeared completely were classified as CR, those whose size remained unchanged as SD, and lesions that increased in size as PD. The initial response assessment was performed after the second or third cycle. Subsequent assessments were conducted at intervals of no more than 3 cycles, depending on the clinical course, with the final assessment performed at the time of treatment discontinuation.

PFS was defined as the time from the initiation of GEMDOX therapy to documented disease progression or death from any cause, which occurred first. OS was measured from the start of GEMDOX therapy to death from any cause. Survivors were censored at the date of their last follow-up. All analyses were performed using IBM SPSS Statistics, Version 22.0 (IBM Corp., Chicago, IL, USA) and *P*-values of <0.05 were considered significant.

## Results

A total of 36 patients were included, and their characteristics are summarized in Table 1. The median age of the patients was 13.5 years (range, 2.0–17.0 years). The most common diagnosis was osteosarcoma (*n* = 21, 58.3%), and 7 (19.4%) patients had metastatic disease at the time of GEMDOX initiation. A median of 6 cycles of GEMDOX were administered per patient (range, 2–12), for a total of 189 cycles. It was most frequently used as a third-line regimen (21 patients, 58.3%), followed by use as a second-line regimen (8 patients, 22.2%) (Table 1). In addition to GEMDOX, 9 (25%) patients received radiotherapy and 13 (36.1%) underwent surgery, while 20 (55.6%) patients received chemotherapy alone.

**Table 1:** Demographic and disease characteristics of the patients.

Characteristic	No (%)
Total no of patients	36
Age at time of treatment with GEMDOX	
Median (yr)	13.5
Range (yr)	2–17
Gender	
Male	17 (47.2)
Female	19 (52.8)
Disease type	
Osteosarcoma	21 (58.3)
Ewing sarcoma family of tumors	6 (16.6)
Rabdomyosarkom	4 (11.1)
Fibrosarcoma	2 (5.5)
Other rare types	3 (8.3)
Stage at time of initial diagnosis	
Localized	29 (80.5)
Metastatic	7 (19.5)
Disease localization	
Extremity	23 (63.9)
Head and neck	6 (16.7)
Chest	3 (8.3)
Abdomen-retroperitoneum	2 (5.6)
Pelvis	2 (5.6)
No of chemotherapy regimens received prior to GEMDOX	
Median (range)	3 (2–5)
2	8 (22.2)
3	21 (58.3)
4	5 (13.9)
5	2 (5.6)
No of GEMDOX courses	
Median (range)	6 (2–12)
Treatments administered during GEMDOX	
Radiotherapy	9 (25.0)
Surgery	13 (36.1)
Only chemotherapy	20 (55.6)

yr, year, GEMDOX, gemcitabine and docetaxel.

**Figure 1:**
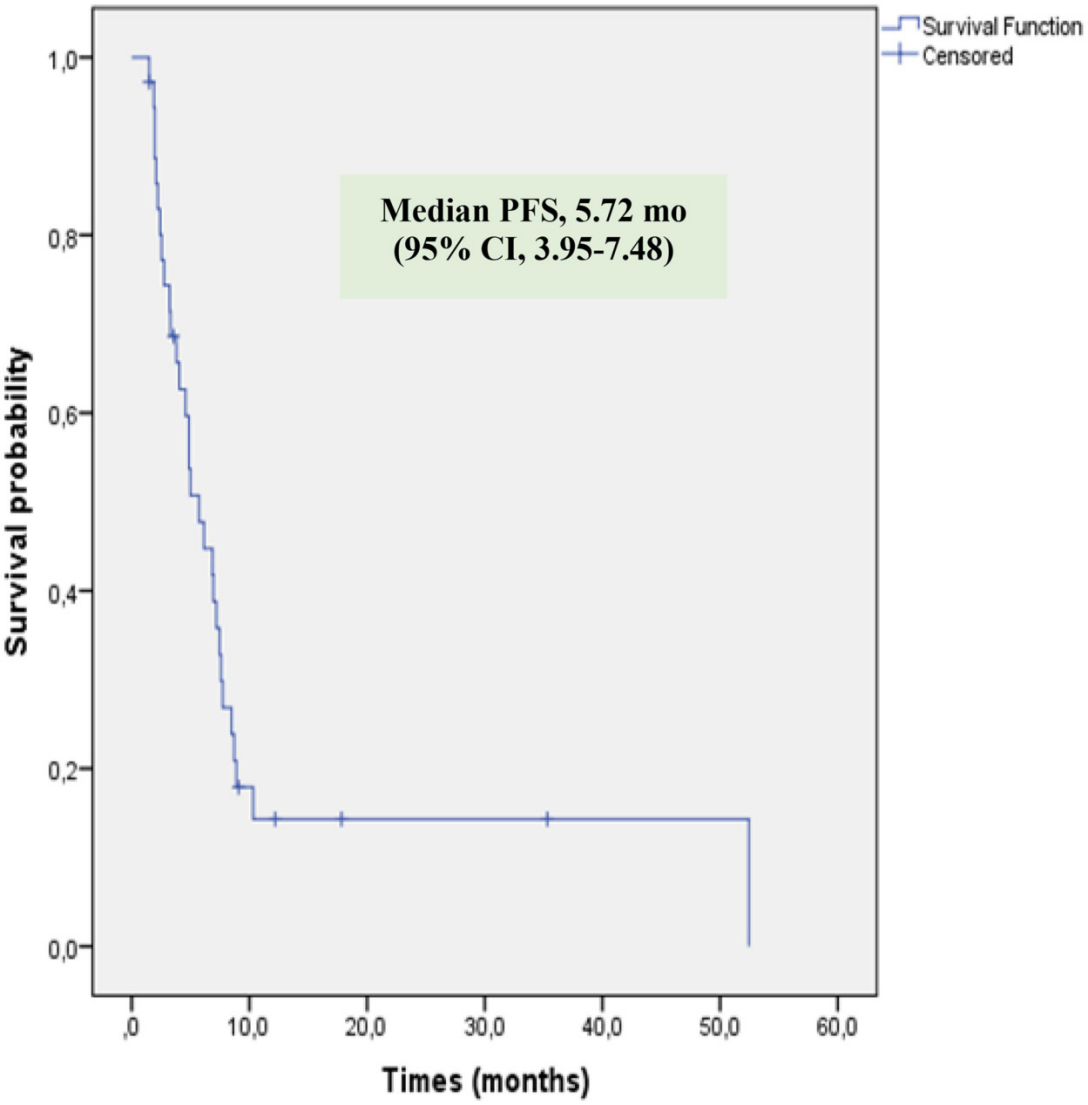
Kaplan–Meier estimates of progresyon-free survival (PFS) in patients.

**Figure 2:**
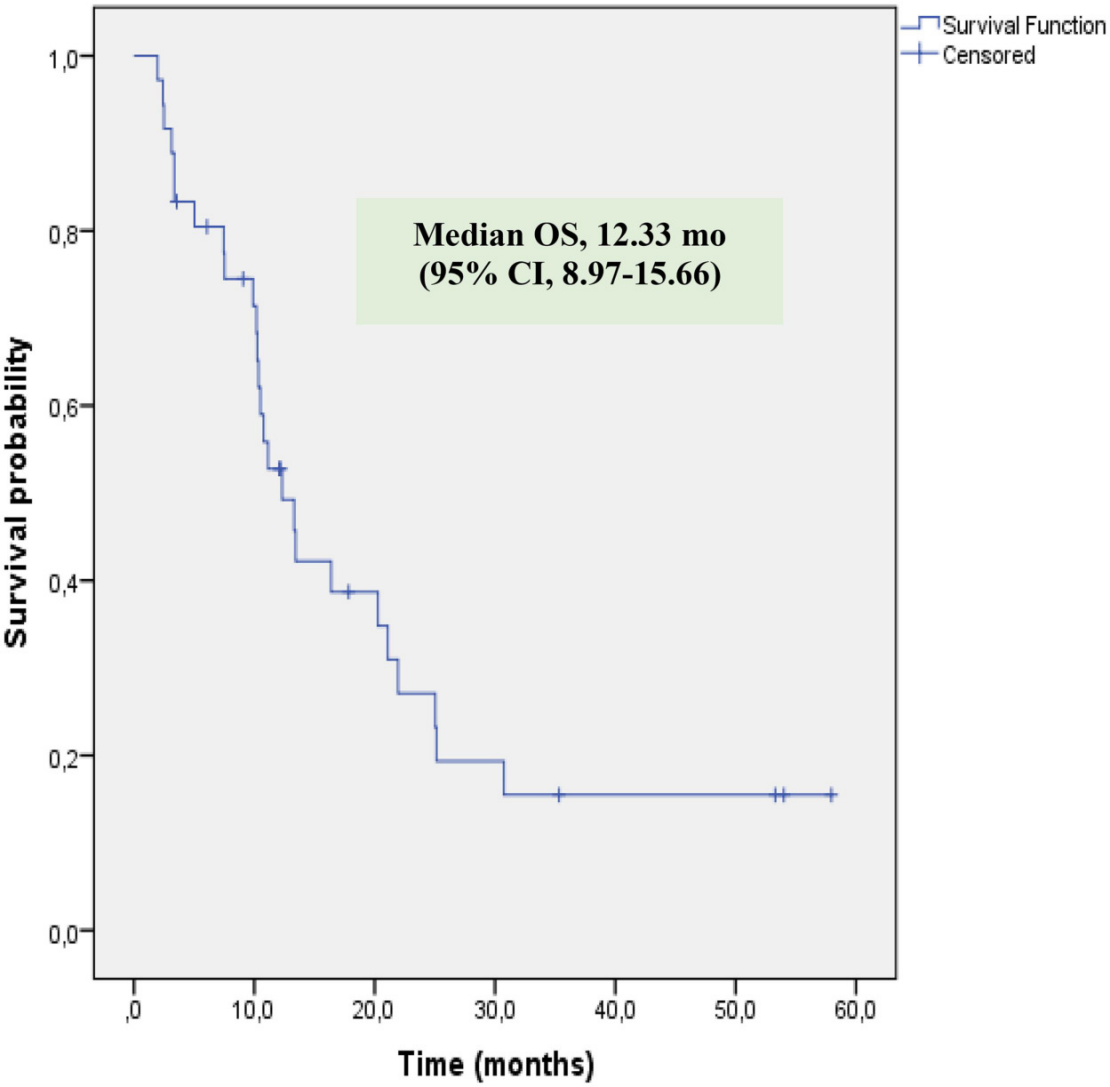
Kaplan–Meier estimates of overall survival (OS) in patients.

Tumor response at the initial evaluation was available for 35 patients (one patient lacked an adequate radiological assessment). Initial tumor responses included complete response (CR) in 2 patients (5.7%), partial response (PR) in 7 patients (20%), stable disease (SD) in 13 patients (37.1%), and progressive disease (PD) in 13 patients (37.1%) (Table 2). The objective response rate (CR + PR) was 25.7%, and disease control (CR + PR + SD) was achieved in 62.8% of patients. At the time of the final response assessment, 34 patients were evaluable. Two patients were excluded from the final efficacy analysis: one patient withdrew consent and refused further treatment, and another discontinued therapy due to a Grade 4 anaphylactic reaction during the third cycle. The patient who experienced anaphylaxis was switched to an alternative chemotherapy regimen. Of this patients, CR was observed in 1 patient (2.9%), PR in 1 patient (2.9%), SD in 3 patients (8.8%), and PD in 29 patients (85.3%). The objective response rate (CR + PR) was 5.8%, and disease control (CR + PR + SD) was achieved in 14.6% of patients. The median PFS and OS durations were 5.72 months [95% confidence interval (CI), 3.95–7.48] and 12.33 months (95% CI, 8.97–15.66), respectively (Figures 1, 2). The 2-year PFS and OS rates were 14.3% and 23.2%, respectively.

**Table 2:** Treatment response and outcome.

Characteristic	No (%)
Initial response evaluation	
Total no of patients	35
Complete response	2 (5.7)
Partial response	7 (20)
Stabil disease	13 (37.1)
Progressive disease	13 (37.1)
Not evaluable	1
Final response evaluation	
Total no of patients	34
Complete response	1 (2.9)
Partial response	1 (2.9)
Stabil disease	3 (8.8)
Progressive disease	29 (85.3)
Treatment refusal	1
Adverse event	1
PFS	
Median (m)	5.72
95% confidence interval (m)	3.95–7.48
OS	
Median (m)	12.33
95% confidence interval (m)	8.97–15.66

M, month; OS, overall survival; PFS, progression-free survival.

Regarding toxicity assessment, 13 patients (36.1%) experienced no adverse events, while 20 patients (55.5%) had mild (Grade 1–2) events. Severe (Grade 3–4) toxicity was observed in 13 patients (36.1%) and no treatment-related mortality occurred. The most frequent severe toxicity was neutropenia (*n* = 8, 22.2%), followed by febrile neutropenia (*n* = 7, 19.4%), thrombocytopenia (*n* = 5, 13.9%), and anemia (*n* = 4, 11.1%) (Table 3). Other severe adverse events, each occurring once, included renal dysfunction, elevated transaminases, and anaphylaxis. Only one patient experienced Grade 4 toxicity (anaphylaxis); all other severe events were Grade 3. Mild (Grade 1–2) toxicities included nausea, electrolyte imbalance, palmar-plantar erythrodysesthesia, and infection. No sensory neuropathy or pulmonary toxicity was observed in any patient (Table 3). A 20% dose reduction was required in only one patient due to febrile neutropenia. At the time of analysis, 9 (25%) patients were alive, 1 (2.8%) was lost to follow-up and 26 (72.2%) had died.

**Table 3:** Treatment-related toxicities.

Adverse event	Grade 1/2 (%)	Grade 3/4 (%)
Neutropenia	6 (16.7)	8 (22.2)
Febrile neutropenia		7 (19.4)
Thrombocytopenia	6 (16.7)	5 (13.9)
Anemia	14 (38.9)	4 (11.1)
Hepatotoxicity	7 (19.4)	1 (2.8)
Nephrotoxicity	3 (8.3)	1 (2.8)
Anaphylaxis^a^		1 (2.8)
Electrolyte imbalance	8 (22.2)	
Infection	4 (11.1)	
Gastrointestinal toxicity	4 (11.1)	
Pulmonary toxicity	1 (2.8)	
Palmar-plantar erythrodysesthesia	1 (2.8)	

a Only grade 4 toxicity observed.

## Discussion

In pediatric solid tumors, particularly sarcomas, for which standard treatment options are limited, survival rates remain below 30% (15). Various treatment strategies and novel agents are being investigated to improve these poor outcomes. We analyzed data from 36 patients with recurrent or refractory sarcoma, who were treated with GEMDOX chemotherapy.

The median age in our cohort was 13.5 years (range, 2.0–17.0), and the most common diagnosis was osteosarcoma (58.3%). Similarly, in a study by Navid et al. evaluating 22 pediatric sarcoma patients, osteosarcoma constituted 77% of cases (11). As osteosarcoma is one of the most common pediatric sarcomas, a high incidence of relapsed/refractory disease is an expected finding (4, 5). Regarding disease localization in our cohort, due to the diagnostic distribution, the extremities were the most frequent site (63.9%), followed by the head and neck (16.7%), which is consistent with the literature (16) There is no standard treatment for relapsed/refractory pediatric sarcomas has led to the use of highly variable chemotherapy protocols. Similar studies report that a different number and treatment lines of chemotherapy regimens were administered, with patients generally receiving a median of 4 cycles (11) A 2025 analysis by the Children's Oncology Group (COG) of relapsed/refractory Non-Rhabdomyosarcoma Soft Tissue Sarcoma (NRSTS) patients confirmed the poor survival and limited salvage options in this population. This therapeutic uncertainty often leads patients to receive multiple lines of therapy (17). In our study, the most of patients (77.8%) received GEMDOX as a third-line chemotherapy regimen (Table 1), and the median number of chemotherapy cycles administered was 6 (range, 2–12). This finding suggests that our patient selection and treatment sequencing are consistent with international series.

The impact of gemcitabine dosage on the efficacy and toxicity profile has been widely discussed in the literature. Specifically, high-dose gemcitabine (900–1,000 mg/m^2^) has been associated with better tumor control and survival in numerous publications (9, 18). The findings in these publications clearly support our decision to use high-dose GEMDOX, as the toxicity profile of high-dose regimens was no greater than low-dose protocols. A study by Lee et al. compared gemcitabine toxicity at doses of 900 mg/m^2^ and 675 mg/m^2^ and reported that hematologic toxicity was lower with the high-dose administration (18). Similarly, Mora et al. studied 10 pediatric patients with relapsed/refractory sarcoma, also using doses of 1,000/100 mg/m^2^, and reported no increase in the toxicity profile (19). Based on the refractory nature of our patients’ disease, literature reports of improved survival, and the lack of increased toxicity, we adopted the 1,000/100 mg/m^2^ dosage in our protocol.

Treatment was generally well-tolerated. In our study, the most frequent adverse event observed during treatment was neutropenia (Table 3). Grade 3 neutropenia occurred in 22.2% (*n* = 8) of patients and Grade 2 in 16.7% (*n* = 6). All patients received granulocyte-colony stimulating factor (G-CSF) therapy for the management of this condition.

Varying approaches to G-CSF administration protocols in the literature. Some studies recommend post-treatment administration, while others suggest its use during treatment (11, 16). In our study, both administration methods (during and after treatment) were utilized, based on the individual clinical and laboratory characteristics of each patient. When compared to the literature, the overall neutropenia rate in our study was markedly lower than the 72.4% severe neutropenia rate reported by Tanaka et al. (16). However, the incidence of febrile neutropenia in our cohort (19.4%) was higher than the 9.7% rate reported in the same study. In another study by Mora et al. evaluating GEMDOX as first-line therapy, the rates of neutropenia and febrile neutropenia were found to be markedly low, at 12% and 2%, respectively (13). The low neutropenia rates in our study suggest the efficacy of our G-CSF protocol, whereas the increased incidence of febrile neutropenia is thought to stem from the specific conditions of our patient population. A significant part of our patients consisted of individuals from low socioeconomic backgrounds who were living in tent cities, where the risk of infection was high, following the major earthquake disaster in our country in 2023. This was considered the most important potential reason for this finding. We believe these adverse living conditions facilitated the development of infections in neutropenic patients, consequently increasing the frequency of febrile neutropenia. Our other adverse event rates were consistent with the literature, and no Grade 4 toxicity was observed, with the exception of anaphylaxis in one patient. The patient who developed anaphylaxis was switched to a different therapeutic regimen. As highlighted by our study and the literature, the manageable toxicity profile of the GEMDOX combination remains a significant advantage (16). However, it is important to acknowledge the associated logistical burden, including the necessity for G-CSF support and potential hospitalizations for febrile neutropenia. While fluid retention and edema are common and clinically adverse effects of docetaxel, no severe (Grade 3–4) cases were observed in our cohort. We attribute this absence of severe toxicity to the effective use of steroid premedication. Furthermore, although not observed in our patient population, severe pulmonary toxicity has been reported in 6.7%–16.9% of cases in other series (16, 20).

Of the 35 patients evaluable for initial response (after at least two cycles), 2 (5.7%) achieved a complete response (CR), 7 (20%) achieved a partial response (PR), 13 (37.1%) had stable disease (SD), and 13 (37.1%) had progressive disease (PD) (Table 2). The objective response rate (ORR; CR + PR) was 25.7%, and the disease control rate (DCR; CR + PR + SD) was achieved in 62.8% of patients. In the literature, treatment response assessments are typically reported at the end of therapy. In our study, however, we also evaluated early treatment responses. The initial response rates appear to be higher than those observed at the final assessment. In such challenging cases, these initial results can be encouraging for the treating physician, as well as for the patient and their family. However, it must be emphasized that in relapsed/refractory patient groups, final response assessments, PFS, and OS are more clinically meaningful indicators of durable efficacy than initial responses, which should be viewed as exploratory. Based on the final analysis of 34 evaluable patients, CR was observed in 1 (2.9%), PR in 1 (2.9%), SD in 3 (8.8%), and PD in 29 (8.5%). This resulted in an objective response rate (ORR; CR + PR) of 5.9% and a disease control rate (DCR; CR + PR + SD) of 14.7%.

The objective response rate in our cohort is low compared to the results of other specific pediatric GEMDOX studies. For instance, Mora et al. reported an ORR of 50%, and Navid et al. reported 29% in their study (11, 19). However, our lower ORR aligns with the results of larger analyses of relapsed/refractory pediatric sarcomas. For example, a comprehensive analysis by Oberoi et al. examined 137 patients with relapsed/refractory NRSTS enrolled in 13 different Phase II studies conducted by the COG. In this analysis, the ORR was reported as only 2.2% (CR 2.2%, PR 0%), with progressive disease observed in over 70% of patients (17). The COG data show that our observed ORR, while low, is consistent with the overall poor response rates reported for this heterogeneous and intensive pretreated population. Several factors may account for the low ORR in our study. First, this may be attributed to the fact that our patient population consisted predominantly of osteosarcoma patients (58.3%), whereas the cohort by Mora et al. was largely composed of patients with Ewing sarcoma (ES) (60%), a histology that may be more sensitive to GEMDOX (19). A second important factor is that most of our patients (77.7%) received the GEMDOX regimen as third-line or later therapy. This finding is consistent with the study by Tanaka et al., which reported an ORR of 18.8% when GEMDOX was used in the first-line setting, compared to an ORR of 6.9% in the second and later lines (16). This finding suggests that the administration of this regimen in later lines of therapy contributes to the lower response rates observed. Finally, sociodemographic factors may also have influenced our outcomes. A significant proportion of our cohort (*n* = 13, 33.3%) consisted of children from Syrian refugee families. Treatment interruptions, related to challenges such as displacement and cross-border travel, were observed in some of these patients. These disruptions in therapy may have compromised treatment adherence and efficacy, thereby impacting the final results.

When analyzing the survival outcomes of our cohort, the median PFS was 5.71 months (95% CI, 3.99–7.43) and the median OS was 12.3 months (95% CI, 8.95–15.68), respectively (Figures 1, 2). The 2-year PFS and OS rates were 14.3% and 23.2%, respectively. Our 2-year OS rate is consistent with the general literature for this challenging patient population. For comparison, the study by Oberoi et al., which we previously cited for its response rates, reported a median OS of only 7 months and a 2-year OS rate of just 16.4% (17) The challenge of treating this population is further highlighted by the findings of Song et al., who reported a 2-year OS rate of 27.8% in their series of 28 relapsed/refractory pediatric sarcoma patients (21). This distinction based on treatment line is emphasized by Tanaka et al., who reported a median OS of 16.4 months in their total cohort, but 22.5 months in the subgroup that received GEMDOX as first-line therapy (16). These data collectively suggest that the use of this regimen in later lines of therapy is a primary reason for our low observed ORR and survival rates. The potential role of GEMDOX in the first-line setting has also been a subject of investigation. A study by Davis et al. evaluated these regimens as a first-line treatment for localized, high-risk soft tissue sarcoma. In that study, the 2-year DFS rate was higher in the GEMDOX arm compared to the doxorubicin and ifosfamide (AI) arm (74% vs. 57%); however, this trend was not statistically significant, and the 2-year OS rates were comparable (91% vs. 87%) (22). Similarly, the Spanish trial (GEIS-21), which investigated GEMDOX as first-line treatment in pediatric patients with newly diagnosed high-risk Ewing sarcoma, reported a 5-year OS rate for high-risk patients of 36.0% (CI, 20%–65%) (13). These results suggest that the regimen's use as a salvage option, typically in later lines, may be a potential reason for our low response rates, and indicate that better outcomes might be achieved if GEMDOX therapy were administered at earlier stages. Considering the dose-intensive nature of GEMDOX, utilizing this regimen earlier before patients become frail or experience excessive toxicity burdens, may maximize both tolerability and therapeutic benefit compared to its use in a late-line palliative setting. However, contrary to these earlier data, the final analysis of the JCOG1306 study was presented at the ASCO 2024 Annual Meeting. These 5-year follow-up results demonstrated a statistically significant lower OS rate for GD compared to the AI regimen (76.1% vs. 90.0%). Therefore, the AI combination maintains its position as the standard-of-care regimen for the first-line treatment of high-risk soft tissue sarcomas (23, 24). Another potential strategy for enhancing the efficacy of GEMDOX has been the addition of Bevacizumab, a vascular endothelial growth factor (VEGF) inhibitor. An example of this approach can be seen in a case series by Hingorani et al., which included three patients with relapsed/refractory pediatric sarcoma. In this study, the addition of Bevacizumab to the GEMDOX regimen resulted in partial responses in two of the three patients and stable disease lasting 6 months in one patient (25). However, due to the very small size of this case series, definitive conclusions cannot be drawn regarding this combination.

For patients with relapsed and refractory sarcoma, several chemotherapy regimens exist beyond GEMDOX. Combinations including irinotecan and temozolomide [with or without vincristine (IT/VIT)], cyclophosphamide and topotecan (TC), and high-dose ifosfamide (IFOS) have demonstrated efficacy (26). In a randomized phase 2/3 study conducted to clarify the efficacy of these regimens, the GEMDOX arm was terminated early following the first interim analysis due to insufficient efficacy compared to the other arms (27). Another important type of salvage regimen is the ifosfamide, carboplatin, and etoposide (ICE) combination. A phase I/II analysis conducted by the Children's Cancer Group (CCG) involving 97 pediatric patients with relapsed/refractory sarcoma (34 with osteosarcoma and 21 with Ewing sarcoma) found that ICE treatment achieved an ORR of 51%, including a CR rate of 27%. The 2-year OS rate in this study was reported as 28% (28) Beyond conventional chemotherapies, novel approaches are also promising. For instance, a phase 1 study that combined Regorafenib with vincristine and irinotecan reported objective responses in pediatric patients with relapsed solid tumors (29).

The efficacy of another targeted agent, the multikinase inhibitor (TKI) pazopanib, was demonstrated in the randomized phase III PALETTE trial conducted by Van der Graaf et al. In this study, pazopanib significantly prolonged PFS compared to placebo (median 4.6 vs. 1.6 months) in patients with metastatic (predominantly adult) non-adipocytic STS who had received prior chemotherapy. However, it did not produce a statistically significant difference in overall survival (30). Although this study predominantly involved an adult population, its findings suggest TKIs, such as Pazopanib, warrant investigation and consideration as potential therapeutic options in pediatric sarcomas.

Our study has several important limitations. First, the patient cohort was histologically heterogeneous; although osteosarcoma comprised the majority (58.3%), the inclusion of various sarcoma subtypes may have led to variability in treatment response and limited our ability to perform statistically meaningful subgroup analyses (osteosarcoma vs. non-osteosarcoma). Second, radiological response was assessed retrospectively based on measurements from existing imaging reports prepared by different radiologists. Furthermore, the administration of GEMDOX in a late-line setting (median: 3rd line) to a heavily pretreated, drug-resistant population likely contributed to our reduced efficacy outcomes. Finally, the single-center design and the relatively small sample size are key factors that limit the generalizability and statistical power of our findings.

The management of relapsed/refractory pediatric sarcoma is complex, and the optimal treatment strategy has not been established. While studies, primarily in adults, show variable efficacy for GEMDOX (31), some pediatric data suggest it can be effective and may offer a more favorable toxicity profile than other chemotherapy regimens (11, 19). The manageability of toxicity with G-CSF, steroids, antiemetic agents, and supportive care plays an important role in this chemotherapy selection (26, 31). The inclusion of only pediatric patients and the relatively large sample size enabled us to conduct our study. Although our data did not demonstrate a survival advantage, we confirmed that GEMDOX has a manageable safety profile. This lack of severe toxicity, particularly due to its acceptable tolerability for patients with poor hematologic reserves, makes this combination a potential salvage option. To clearly define its role, further prospective studies are warranted, particularly by utilizing the combination in earlier lines of therapy and comparing it against other high-risk protocols.

## Data Availability

The raw data supporting the conclusions of this article will be made available by the authors, without undue reservation.
